# Different associations between body mass index and Alzheimer’s markers depending on metabolic health

**DOI:** 10.1186/s13195-024-01563-z

**Published:** 2024-08-29

**Authors:** Eun Hye Lee, Heejin Yoo, Young Ju Kim, Bo Kyoung Cheon, Seungho Ryu, Yoosoo Chang, Jihwan Yun, Hyemin Jang, Jun Pyo Kim, Hee Jin Kim, Seong-Beom Koh, Jee Hyang Jeong, Duk L. Na, Sang Won Seo, Sung Hoon Kang

**Affiliations:** 1 Department of Neurology, Samsung Medical Center, Sungkyunkwan University School of Medicine, 81 Irwon-ro, Gangnam-gu, Seoul, 06351 Republic of Korea; 2 Alzheimer’s Disease Convergence Research Center, Samsung Medical Center, Seoul, Republic of Korea; 3 Center for Cohort Studies, Total Healthcare Center, Kangbuk Samsung Hospital, Sungkyunkwan University School of Medicine, Seoul, Republic of Korea; 4 Department of Neurology, Soonchunhyang University Bucheon Hospital, Gyeonggi-do, Republic of Korea; 5 Department of Neurology, Seoul National University Hospital, Seoul National University college of Medicine, Seoul, Republic of Korea; 6 Department of Digital Health, SAIHST, Sungkyunkwan University, Seoul, Republic of Korea; 7 Department of Health Sciences and Technology, SAIHST, Sungkyunkwan University, Seoul, Republic of Korea; 8 Department of Neurology, Korea University Guro Hospital, Korea University College of Medicine, 148 Gurodong-ro, Guro-gu, Seoul, 08308 Republic of Korea; 9 Department of Neurology, Ewha Womans University Seoul Hospital, Ewha Womans University College of Medicine, Seoul, Republic of Korea; 10 Department of Intelligent Precision Healthcare Convergence, Sungkyunkwan University, Suwon, Republic of Korea

**Keywords:** Obesity, Underweight, Metabolic health, MHO, Alzheimer’s disease, Biomarkers

## Abstract

**Background:**

Increasing evidence supports the association between body mass index (BMI), Alzheimer’s disease, and vascular markers. Recently, metabolically unhealthy conditions have been reported to affect the expression of these markers. We aimed to investigate the effects of BMI status on Alzheimer’s and vascular markers in relation to metabolic health status.

**Methods:**

We recruited 1,736 Asians without dementia (71.6 ± 8.0 years). Participants were categorized into underweight, normal weight, or obese groups based on their BMI. Each group was further divided into metabolically healthy (MH) and unhealthy (MU) groups based on the International Diabetes Foundation definition of metabolic syndrome. The main outcome was Aβ positivity, defined as a Centiloid value of 20.0 or above and the presence of vascular markers, defined as severe white matter hyperintensities (WMH). Logistic regression analyses were performed for Aβ positivity and severe WMH with BMI status or interaction terms between BMI and metabolic health status as predictors. Mediation analyses were performed with hippocampal volume (HV) and baseline Mini-Mental State Examination (MMSE) scores as the outcomes, and linear mixed models were performed for longitudinal change in MMSE scores.

**Results:**

Being underweight increased the risk of Aβ positivity (odds ratio [OR] = 2.37, 95% confidence interval [CI] 1.13–4.98), whereas obesity decreased Aβ positivity risk (OR = 0.63, 95% CI 0.50–0.80). Especially, obesity decreased the risk of Aβ positivity (OR = 0.38, 95% CI 0.26–0.56) in the MH group, but not in the MU group. Obesity increased the risk of severe WMH (OR = 1.69, 1.16–2.47). Decreased Aβ positivity mediate the relationship between obesity and higher HV and MMSE scores, particularly in the MH group. Obesity demonstrated a slower decline in MMSE (β = 1.423, *p* = 0.037) compared to being normal weight, especially in the MH group.

**Conclusions:**

Our findings provide new evidence that metabolic health has a significant effect on the relationship between obesity and Alzheimer’s markers, which, in turn, lead to better clinical outcomes.

**Supplementary Information:**

The online version contains supplementary material available at 10.1186/s13195-024-01563-z.

## Background

Inceasing epidemiological evidence supports the association between body mass index (BMI) and Alzheimer’s disease (AD) markers. Specifically, obesity in late life is associated with low amyloid beta (Aβ) accumulation, while being underweight poses a high risk for Aβ burden [1–3]. Moreover, studies conducted by our research group have shown that metabolically unhealthy conditions, including hypertension [4], diabetes mellitus [5], and dyslipidemia [6], are associated with increased AD markers including Aβ uptakes, hippocampal atrophy, and cognitive decline in non-dementia participants. Considering that obesity and the metabolically unhealthy conditions may have opposing effects on AD markers, we hypothesized that the protective effects of obesity on AD markers may be more prominent in the metabolically healthy (MH) group than in the metabolically unhealthy (MU) group. We also hypothesized that the detrimental effects of being underweight on AD markers may be more pronounced in the MU group than in the MH group. However, no differences may be observed in the relationship between obesity and cerebral small vascular disease (CSVD) markers based on metabolic health, as BMI has been shown to exhibit a U-shaped risk profile with regard to cardiovascular diseases [7, 8]. CSVD markers including white matter hyperintensities (WMH) commonly occur in AD [9]. However, most previous studies did not take the MH status into account when assessing the effect of BMI status on AD and CSVD markers [10–12]. Furthermore, previous studies have included some participants with dementia, despite the fact that the effects of BMI status on AD markers may differ between non-dementia and dementia stages [3].

In this study, we aimed to investigate the effects of BMI on AD and CSVD markers, as well as their clinical implications in relation to metabolic health, in a large Asian cohort without dementia. First, we sought to determine whether being underweight or obese was associated with Aβ positivity on positron emission computed tomography (PET) scans, with a specific focus on how these associations varied with metabolic health status. Second, we aimed to investigate whether the BMI statuses were associated with severe WMH, again considering the metabolic health status. Third, we examined whether statistically significant markers from previous analyses mediate the relationship between BMI, hippocampal atrophy, and cognitive impairment, especially in the MH group. Finally, we explored the effects of BMI on longitudinal cognitive decline according to metabolic health status.

## Methods

### Study participants

We recruited a total of 1,772 participants who were either cognitively unimpaired (CU) or had a mild cognitive impairment (MCI) and underwent an Aβ PET scan at the Samsung Medical Center between August 2015 and January 2023. The CU participants were composed of spouses of patients who visited the memory clinic, volunteers who applied for comprehensive dementia evaluation advertised in the paper, and participants who had cognitive complaints. The diagnostic criteria for CU were as follows: (1) no medical history that could potentially affect cognitive function based on Christensen’s health screening criteria [13]; (2) no objective cognitive impairment in any cognitive domain, as determined by a comprehensive neuropsychological test battery (performance above at least −1.0 standard deviation [SD] of age-adjusted norms on any cognitive test); and (3) independence in activities of daily living. Detailed information of the neuropsychological test battery is described in Supplementary Methods. The criteria for diagnosing MCI were based on the 2011 National Institute on Aging-Alzheimer’s Association Diagnostic Guidelines [14].

All participants underwent clinical interviews, neurological and neuropsychological examinations, and laboratory tests, including complete blood count, blood chemistry, thyroid function tests, syphilis serology, and vitamin B_12_/folate levels. The absence of structural lesions, including cerebral infarctions, brain tumors, vascular malformations, and hippocampal sclerosis, was confirmed using brain magnetic resonance imaging (MRI).

This study was approved by the Institutional Review Board of the Samsung Medical Center. Written informed consent was obtained from all participants prior to their participation.

### BMI status

**Fig. 1:**
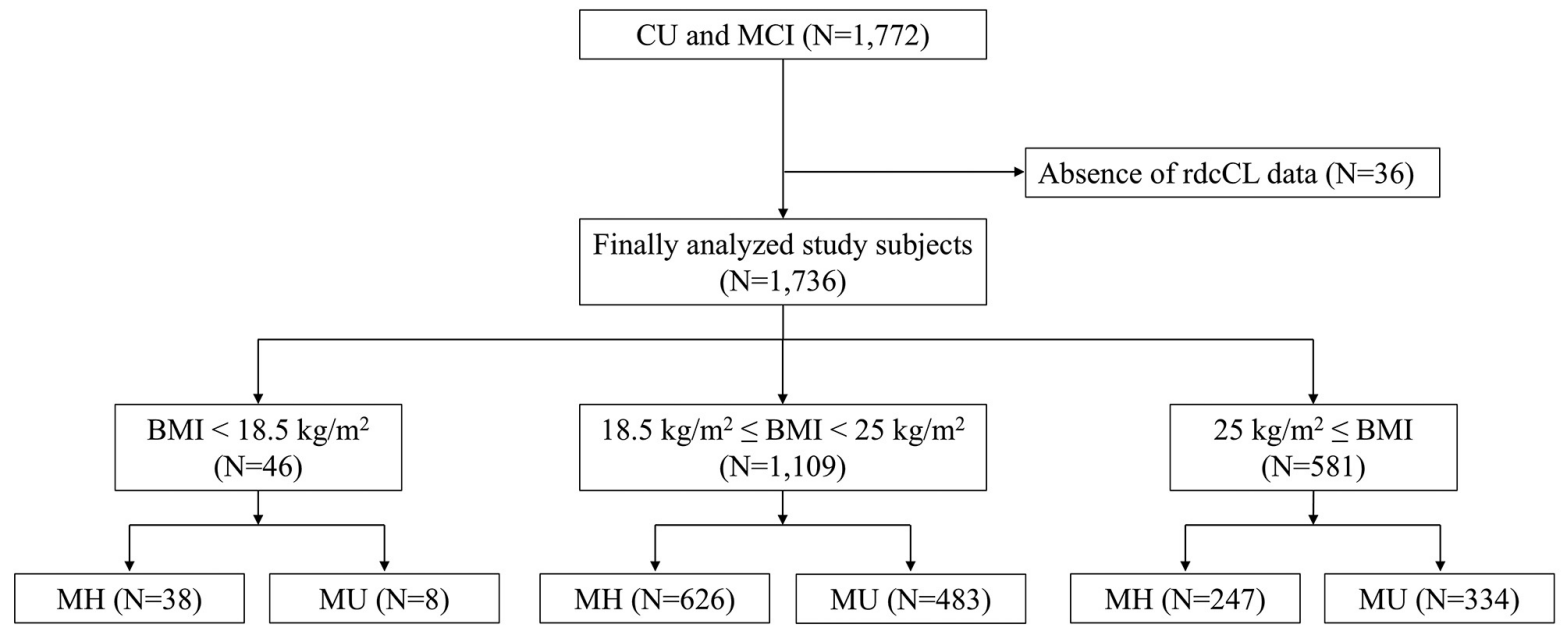
Flow of participant selection. A total of 1,772 participants with CU or MCI were recruited for this study. After excluding 36 participants without centiloid data from Aβ PET, the final analyses included 1,736 participants. Participants were sorted into three groups based on their BMI: BMI below 18.5 kg/m^2^ as underweight, BMI between 18.5 kg/m^2^ and 24.9 kg/m^2^ as normal weight group, BMI above 25 kg/m^2^ as obesity group. Subsequently, participants in each BMI status group were classified into the MH and MU groups according to theInternational Diabetes Foundation definition of metabolic syndrome and previous studies. BMI = body mass index; CU = cognitively unimpaired; MCI = mild cognitive impairment; MH = metabolically healthy; MU = metabolically unhealthy; rdcCL = regional direct comparison centiloid

According to the World Health Organization, obesity phenotypes in Asians are categorized by BMI [15]: below 18.5 kg/m^2^ as underweight, between 18.5 kg/m^2^ and 23 kg/m^2^ as normal weight, between 23 kg/m^2^ and 25 kg/m^2^ as overweight and above 25 kg/m^2^ as obese. In this study, we grouped participants between 18.5 kg/m^2^ and 25 kg/m^2^ as one group because our previous research has shown that individuals in this range of BMI have similar patterns of AD markers [16–18]. Thus, in this study, participants were divided into three groups as follows: below 18.5 kg/m^2^ as underweight, between 18.5 kg/m^2^ and 24.9 kg/m^2^ as normal weight, and above 25 kg/m^2^ as obese (Fig. 1).

### Definitions of metabolic health

Participants were also divided into MH and MU groups using criteria based on the International Diabetes Foundation (IDF) definition of metabolic syndrome and previous studies (Fig. 1) [19–22]. The waist circumference criterion from the IDF definition was not included because of its collinearity with BMI. Participants were classified into the MU group if they met two or more of the criteria: (1) elevated systolic blood pressure (≥ 130 mmHg) or diastolic blood pressure (≥ 85 mmHg) or receiving antihypertensive treatment; (2) elevated fasting plasma glucose (≥ 100 mg/dL) or receiving antidiabetic treatment; (3) elevated triglycerides (≥ 150 mg/dL) or receiving specific treatment for triglycerides abnormality; and (4) reduced high-density lipoprotein cholesterol (HDL-C) levels (< 40 mg/dL in men and < 50 mg/dL in women) or receiving specific treatment for HDL-C abnormality.

### Aβ PET acquisition and quantification

In order to measure Aβ deposition which is one of the earliest recognizable pathological events in Alzheimer’s disease [23], all participants underwent Aβ PET scans using a Discovery STe PET/CT scanner (GE Medical Systems, Milwaukee, WI, USA) with either ^18^F-florbetaben (FBB) or ^18^F-flutemetamol (FMM). PET scans were performed in dynamic mode including four 5-min frames, which resulted in a 20-min emission scan. Scans were performed 90 min after the injection of an average dose of 311.5 MBq FBB or 197.7 MBq FMM. The detailed imaging acquisition protocols are described in Supplementary Methods.

We used the regional direct comparison centiloid (rdcCL) method to conduct the normalized quantitative analysis of PET-measured Aβ [24]. The detailed quantification methods are described in Supplementary Methods. We defined Aβ positivity using a global rdcCL cutoff value of 20.0, which has been increasingly used as a criterion in various cohort studies and clinical trials [25, 26]. Thirty-six participants without rdcCL data from Aβ PET were excluded. Thus, the final analysis included 1,736 participants.

### MRI acquisition and quantification

We acquired standardized three-dimensional T1 Turbo Field Echo and three-dimensional fluid-attenuated inversion recovery (FLAIR) images using a 3.0 T MRI scanner (Philips 3.0T Achieva; Philips Healthcare, Andover, MA, USA), as previously described [27].

Hippocampal atrophy is a recognized biomarker of neurodegeneration in Alzheimer’s disease, as suggested by the National Institute on Aging-Alzheimer’s Association [28–30] and the National Institute of Neurological Disorders and Stroke–Alzheimer Disease and Related Disorders working groups [31]. To measure the hippocampal volume (HV), we used an automated hippocampal segmentation method using a graph-cut algorithm combined with atlas-based segmentation and morphological opening, as described in a previous study [32].

WMH severity was defined using the WMH visual rating scale, proposed by the Clinical Research Center for Dementia in South Korea (CREDOS). According to our previous study, the presence of severe WMH indicates the severity of CSVD markers, including WMH volume, number of lacunes, and number of microbleeds [33]. Severe WMH was defined based on the following criteria: (1) WMH of 10 mm or more in the periventricular white matter (caps or rim) and (2) WMH of 25 mm or more (maximum diameter) in the deep white matter, consistent with an extensive white matter lesion or diffusely confluent lesion [27]. WMH severity was manually evaluated by the experienced neurologists. The inter-rater reliability of the CREDOS WMH visual rating scale was previously found to be excellent (intra-class correlation coefficient between 0.726 and 0.905) [34].

### Longitudinal assessment of cognitive decline

The Mini-Mental State Examination (MMSE) [35] was used to assess global cognition at and throughout the assessment of longitudinal cognitive decline because MMSE has long been the most widely used tool for screening and follow-up of cognitive function. Among the 1,736 participants, a total of 1,723 participants underwent follow-up MMSE assessments. The mean assessment period was 3.1 ± 3.5 years, and the number of MMSE assessments was 3.1 ± 0.1.

### Statistical analyses

Demographic and clinical characteristics are presented as mean (SD) for continuous variables and as numbers (percentages) for categorical variables.

To investigate the impact of BMI status on Aβ positivity, logistic regression was conducted using BMI status as the predictor and Aβ positivity as the outcome after controlling for age, sex, *APOE* genotype (ε4 non-carrier vs. ε4 carrier), and disease stage (CU vs. MCI). We also performed logistic regression using the same model for each metabolic health status group, along with a logistic regression that included an interaction term between BMI status and metabolic health status in the entire study population.

To investigate the impact of BMI on severe WMH, logistic regression analysis was conducted after controlling for age, sex, and disease stage. We also performed logistic regression using the same model for each metabolic health status group, along with a logistic regression that included an interaction term between BMI status and metabolic health status in the entire study population.

To determine whether AD and CSVD markers that were significantly associated with BMI status affected clinical outcomes (HV and MMSE), we performed a mediation analyses. Prior to analysis, we confirmed that HV and MMSE satisfied the assumptions of normal distribution [36]. To identify whether Aβ positivity mediated the effect of BMI status on HV and MMSE score, we performed mediation analyses, after controlling for age, sex, *APOE* genotype, and disease stage. Intracranial volume and years of education were added as covariates for HV and MMSE scores, respectively. Bootstrapping was used to verify the significance of indirect effects. To identify whether severe WMH mediated the effect of BMI status on HV and MMSE score, we performed mediation analyses after controlling for age, sex, and disease stage. *APOE* genotype was controlled as a covariate for both HV and MMSE scores, while intracranial volume and years of education were added as covariates for HV and MMSE scores, respectively.

To investigate the effects of BMI status on longitudinal MMSE scores, we performed a linear mixed model after including age, sex, *APOE* genotype, disease stage, years of education, and the interaction term between time and BMI status (time × BMI status) as covariates. The interaction between BMI and metabolic health was assessed using a three-way interaction term (time × BMI status × metabolic health status). Due to a skewed distribution of longitudinal MMSE data, the MMSE scores for was transformed using a Box-Cox transformation [37] prior to its inclusion in linear mixed model analyses.

Covariates known to significantly affect the outcome variable were used in all analyses. *APOE* genotype, which is known to influence Aβ positivity, HV, and cognition, was adjusted for when these variables were outcomes. However, because its association with WMH is not well established, *APOE* genotype was not adjusted for when severe WMH was the outcome. Similarly, education, which has little or inconsistent effects on Aβ positivity, severe WMH, and HV but is strongly correlated with cognition, was used as a covariate only when the MMSE was the outcome.

All reported *p*-values were two-tailed and the significance level was set at 0.05. When conducting analyses within each healthy group, we applied multiple comparison corrections using the Bonferroni correction method. All the analyses were performed using using R version 4.2.3 (The R Foundation for Statistical Computing, Vienna, Austria), SAS version 9.4 (SAS Institute Inc., Cary, NC, USA) and Mplus version 8.1 (Muthén & Muthén, LA, CA, USA).

## Results

### Participants’ clinical characteristics

The demographic and clinical characteristics of the study participants are shown in Table 1. The mean age, along with standard deviations (± SD) was 71.6 ± 8.0 years, and 1,015 (58.5%) participants were female. Further, 591 (34.0%) participants were CU. Of the total 1,736 participants, 46 (2.6%) were categorized into the underweight group, 1,109 (63.9%) into the normal weight group, and 581 (33.5%) into the obese group. Regarding metabolic health status, 38 (82.6%) MH participants were in the underweight group, 626 (56.4%) in the normal weight group, and 247 (42.5%) in the obese group.

**Table 1:** Baseline characteristics according to BMI status and metabolic health status

	Total (*N* = 1,736)	BMI < 18.5 kg/m^2^ (*N* = 46, 2.6%)	18.5 kg/m^2 ^≤ BMI < 25 kg/m^2^ (*N* = 1109, 3.9%)	25 kg/m^2 ^≤ BMI (*N* = 581, 33.5%)	MH (*N* = 911, 52.5%)			MU (*N* = 825, 47.5%)		
					BMI < 18.5 kg/m^2^ (*N* = 38, 4.2%)	18.5 kg/m^2 ^≤ BMI < 25 kg/m^2 ^ (*N* = 626, 68.7%)	25 kg/m^2 ^≤ BMI (*N* = 247, 27.1%)	BMI < 18.5 kg/m^2^ (*N* = 8, 1.0%)	18.5 kg/m^2^ ≤ BMI < 25 kg/m^2^ (*N* = 483, 58.5%)	25 kg/m^2 ^≤ BMI (*N* = 334, 40.5%)
**Demographics**										
Age, years	71.6 ± 8.0	71.8 ± 9.0	71.4 ± 8.0	71.8 ± 7.8	70.7 ± 9.2*	70.3 ± 8.3	70.5 ± 8.1*	76.6 ± 6.2	72.9 ± 7.4	72.8 ± 7.5
Sex, female	1,015 (58.5%)	40 (87.0%)*	668 (60.2%)	307 (52.8%)*	33 (86.8%)	393 (62.8%)	130 (52.6%)	7 (87.5%)	275 (56.9%)	177 (53.0%)
Education, years	12.1 ± 4.7	12.5 ± 4.8	12.3 ± 4.6	11.8 ± 4.9	13.1 ± 4.5	12.5 ± 4.6	12.0 ± 4.7	10.0 ± 5.6	11.9 ± 4.6	11.7 ± 5.0
*APOE*, ε4 carrier	606 (34.9%)	25 (54.3%)*	399 (36.0%)	182 (31.3%)	20 (52.6%)*	221 (35.3%)	68 (27.5%)*	5 (62.5%)	178 (36.9%)	114 (34.1%)
Disease stage, MCI	1,145 (66.0%)	34 (73.9%)	723 (65.2%)	388 (66.8%)	29 (76.3%)	391 (62.5%)	165 (66.8%)	5 (62.5%)	332 (68.7%)	223 (66.8%)
**ATP-III component** ^a^										
Elevated BP	1,190 (68.5%)	27 (58.7%)	717 (64.7%)	446 (76.8%)*	19 (50.0%)	291 (46.5%)	143 (57.9%)*	8 (100.0%)	426 (88.2%)	303 (90.7%)
Elevated FPG	761 (43.8%)	10 (21.7%)*	476 (42.9%)	275 (47.3%)	5 (13.2%)	86 (13.7%)	29 (11.7%)	5 (62.5%)	390 (80.7%)	246 (73.7%)*
Elevated TG	373 (21.5%)	3 (6.5%)*	221 (19.9%)	149 (25.6%)*	1 (2.6%)	30 (4.8%)	11 (4.5%)	2 (25.0%)	191 (39.5%)	138 (41.3%)
Reduced HDL	307 (17.7%)	3 (6.5%)	175 (15.8%)	129 (22.2%)*	1 (2.6%)	15 (2.4%)	7 (2.8%)	2 (25.0%)	160 (33.1%)	122 (36.5%)
**Dementia markers**										
Aβ positivity^b^	769 (44.3%)	34 (73.9%)*	520 (46.9%)	215 (37.0%)*	28 (73.7%)*	303 (48.4%)	73 (29.6%)*	6 (75.0%)	217 (44.9%)	142 (42.5%)
Severe WMH^c^	129 (7.4%)	3 (6.5%)	68 (6.1%)	58 (10.0%)*	3 (7.9%)	18 (2.9%)	13 (5.3%)	0 (0.0%)	50 (10.4%)	45 (13.5%)
**Clinical outcomes**										
Hippcampa volume, mm^3^	2786.0 ± 521.6	2428.0 ± 592.9*	2787.7 ± 515.6	2812.2 ± 516.8	2820.7 ± 517.2*	2884.7 ± 532.2	2457.7 ± 641.8	2744.5 ± 510.9*	2759.3 ± 499.4	2287.2 ± 235.7
MMSE raw score	26.5 ± 3.1	25.8 ± 3.5	26.5 ± 3.1	26.5 ± 3.0	26.8 ± 2.9	26.6 ± 2.9	26.0 ± 3.3	26.1 ± 3.3	26.4 ± 3.0	24.5 ± 4.5

Unless otherwise noted, values are expressed as N (%) or mean ± standard deviation

*Significantly different values compared to normal weight in the total population and in each metabolic health group. P-values for continuous variables were obtained using the Student’s t-test, while for categorical variables they were calculated using chi-square tests

^a^Each ATP-III component was defined as follows: elevated BP, systolic BP ≥ 130 mmHg or diastolic BP ≥ 85 mmHg or receiving antihypertensive treatment; elevated FPG, FPG ≥ 100 mg/dL or receiving antidiabetic treatment; elevated TG, TG ≥ 150 mg/dL; reduced HDL, HDL-C levels < 40 mg/dL in men and < 50 mg/dL in women

^b^Aβ positivity was defined by an amyloid PET Centiloid value greater than 20.0

^c^Severe WMH was defined by the following criteria: (1) WMH of 10 mm or more in the periventricular white matter (caps or rim) and (2) WMH of 25 mm or more (maximum diameter) in the deep white matter, consistent with an extensive white matter lesion or a diffusely confluent lesion

*Aβ* amyloid beta, *BMI* body mass index, *BP* blood pressure, *CU* cognitively unimpaired, *DM* diabetes mellitus, *FPG* fasting plasma glucose, *HDL* high density lipoprotein cholesterol, *HTN* hypertension, *MMSE* mini-mental state exam, *MCI* mild cognitive impairment, *MH* metabolically healthy, *MU* metabolically unhealthy, *TG* triglyceride, *WMH* white matter hyperintensities

### Effects of BMI on Alzheimer’s and vascular markers based on metabolic health

**Fig. 2:**
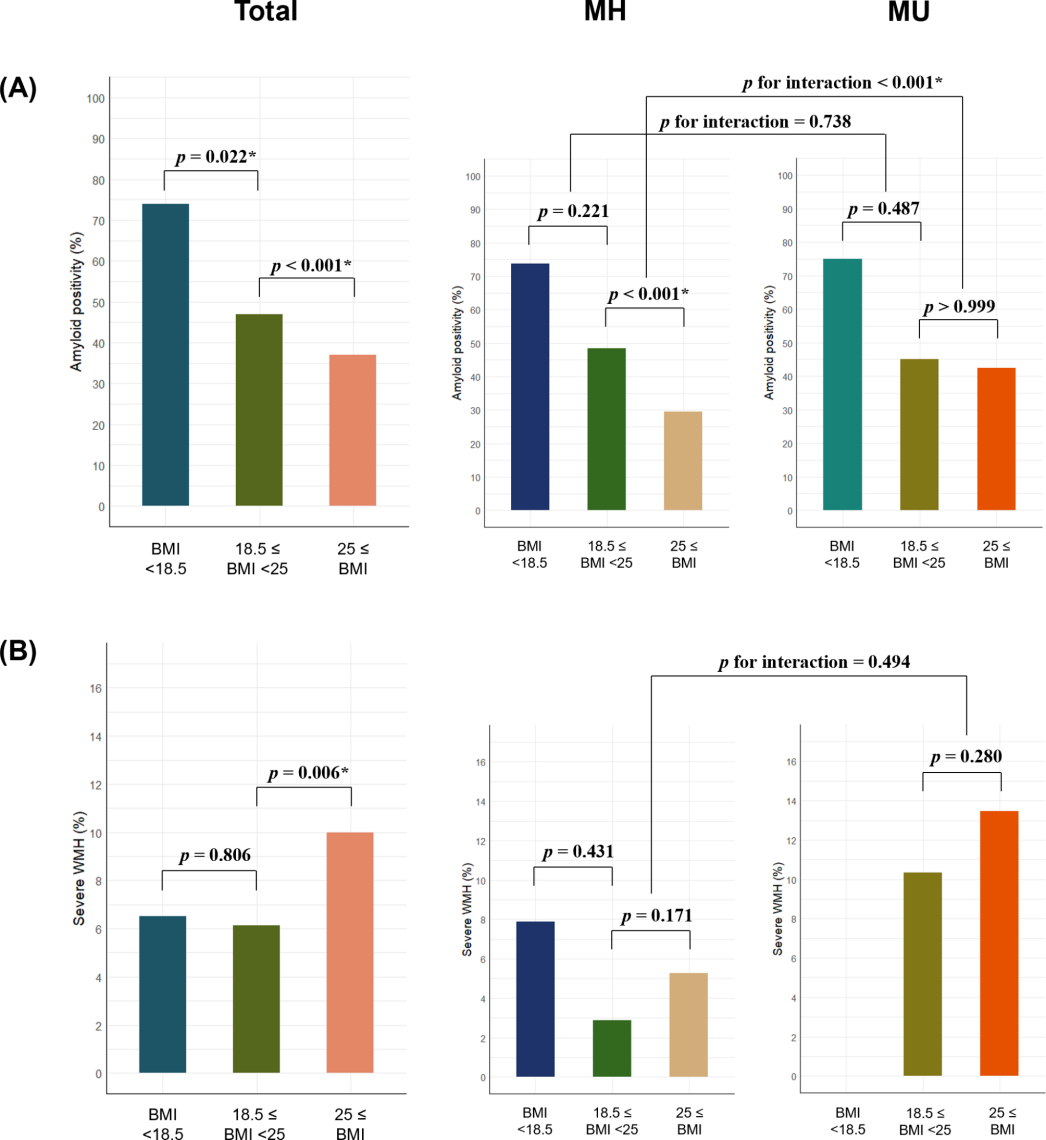
Effect of metabolic health on the association between BMI status and Aβ positivity and severe WMH. (**A**) The risk of Aβ positivity increased in the underweight group but decreased in obesity group. Obesity decreased the risk of Aβ positivity in the MH group. Obesity did not increase the risk of Aβ positivity in the MU group. There was an interaction between obesity and metabolic health on Aβ positivity. (**B**) Obesity was associated with a higher risk of severe WMH. No significant interaction was observed between obesity and metabolic health on severe WMH. BMI = body mass index; MH = metabolically healthy; MU = metabolically unhealthy; WMH = white matter hyperintensity

Figure 2A shows an increased risk of Aβ positivity among individuals who are underweight (odds ratio [OR] = 2.37, 95% confidence interval [CI] 1.13–4.98). In contrast, obesity was associated with a decreased risk of Aβ positivity (OR = 0.63, 95% CI 0.50–0.80). Notably, within the MH group, obesity significantly decreased the risk of Aβ positivity (OR = 0.38, 95% CI 0.26–0.56), while in MU group it did not (OR = 0.97, 95% CI 0.70–1.33). These results indicate an interaction between obesity and metabolic health status concerning Aβ positivity (*p* for obesity [reference: normal weight] × metabolic health status [reference: MH] < 0.001).

Figure 2B shows that obesity was associated with a higher risk of severe WMH (OR = 1.69, 95% CI 1.16 to 2.47, *p* = 0.006). Further, no significant interaction was observed between obesity and metabolic health in patients with severe WMH (*p* for obesity × metabolic health status = 0.494).

### Mediation between BMI status and clinical outcomes

**Fig. 3:**
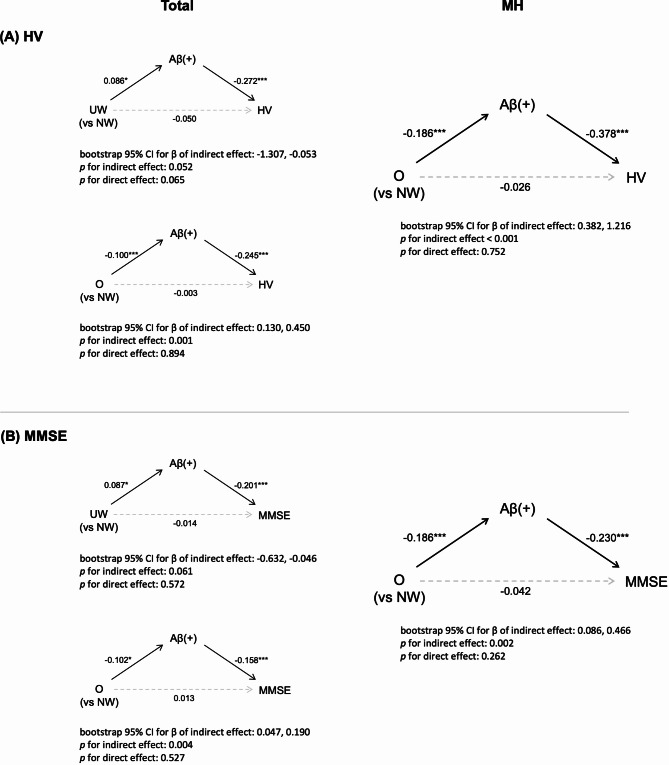
Mediation analysis via amyloid-mediated pathways. In this study, UW was defined as a BMI less than 18.5 kg/m^2^, NW was defined as between 18.5 kg/m^2^ and 24.9 kg/m^2^, and O was defined as a BMI greater than 25 kg/m^2^. Statistically significant associations are expressed as solid lines, whereas non-significant associations are indicated by dashed lines. β value for each association are written on the line. (**A**) Aβ positivity fully mediated the association of BMI status with HV in the whole population. In the MH group, obesity was associated with higher HV only mediated by the absence of Aβ positivity. (**B**) The association of BMI status with MMSE was also fully mediated by Aβ positivity in the total population. Obesity was associated with higher MMSE, and only mediated by the absence of Aβ positivity in the MH group. Aβ(+) = amyloid beta positivity; HV = hippocampal volume; MMSE = mini-mental state exam; NW = normal weight group; O = obesity; UW = underweight

The presence of Aβ positivity fully mediated the association between being underweight and lower HV (Fig. 3A) as well as lower MMSE scores (Fig. 3B). The absence of Aβ positivity also fully mediated the association between obesity and higher HV (Fig. 3A) as well as higher MMSE scores (Fig. 3B). Especially, in the MH group, the absence of Aβ positivity also fully mediated the association between obesity and higher HV (Fig. 3A) as well as higher MMSE scores (Fig. 3B).

The presence of WMH did not mediate the relationship between obesity HV and MMSE scores (Supplementary Fig. 1).

### Effect of BMI on longitudinal MMSE change by metabolic health

**Fig. 4:**
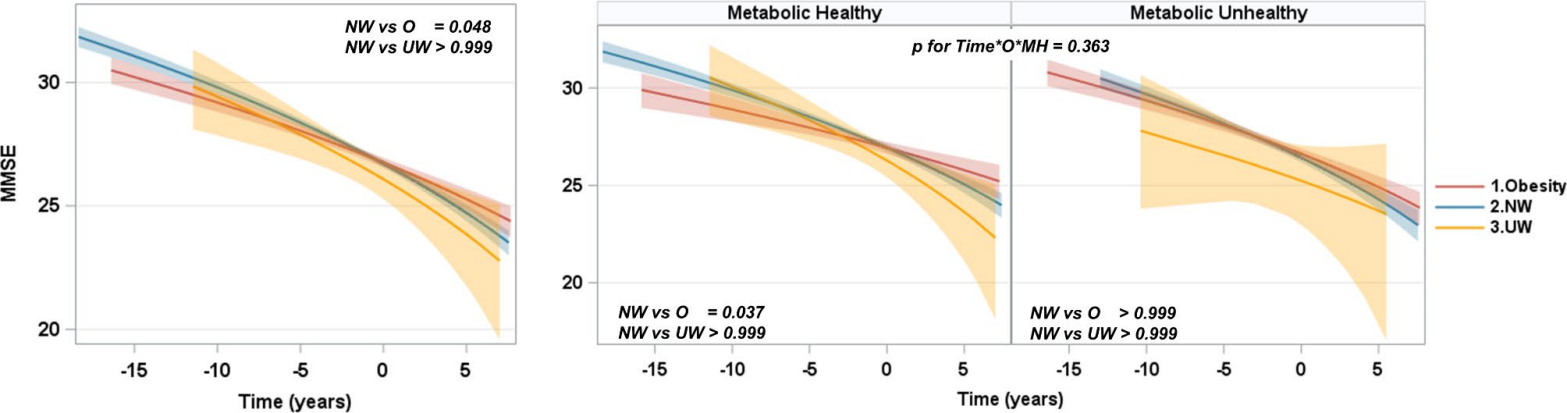
Longitudinal MMSE changes by BMI status and metabolic health. In this study, UW was defined as a BMI less than 18.5 kg/m2, NW was defined as a BMI between 18.5 kg/m^2^ and 24.9 kg/m^2^, and O was defined as a BMI greater than 25 kg/m^2^. The analyses were conducted using Box-Cox transformed longitudinal MMSE data. The predicted values from the fitted model were retransformed to the raw MMSE scale and utilized to plot the graph. (**A**) The underweight group showed no significant difference in MMSE changes over time, whereas the obese group showed a slower decline in MMSE scores compared to the normal weight group. (**B**) The rate of MMSE score decline was slower in the obese group than in the normal-weight group, but the interaction between BMI status and metabolic health was not statistically significant. MMSE, Mini-Mental State Examination; NW, normal weight group; O, obese; UW, underweight; WMH, white matter hyperintensity

Being underweight showed no significant difference in MMSE changes over time (β = 0.621, *p* > 0.999), whereas obesity was associated with a slower decline in MMSE (β = 0.793, *p* = 0.048) when compared to the normal weight group (Fig. 4A). Notably, within the MH group, the rate of MMSE score decline was slower in the presence of obesity compared to individuals with a normal weight (β = 1.423, *p* = 0.037) (Fig. 4B). No interaction between obesity and metabolic health was observed in the MMSE score decline over time (*p* for time × obesity × metabolic health status = 0.363).

### Sensitivity analyses

When the participants were stratified by *APOE* genotype (ε4 non-carrier vs. ε4 carrier), both stratified groups showed significant associations between obesity and decreased risk of Aβ positivity only in the MH group (ε4 non-carrier: OR = 0.41, 95% CI 0.24–0.70; ε4 carrier: OR = 0.32, 95% CI 0.15–0.70) (Supplementary Fig. 2). There were no statistical significances in the three-way interaction (*p* for *APOE* genotype × obesity × metabolic health status = 0.821).

When stratifying participants by disease stage (CU and MCI), both CU and MCI groups revealed significant associations between obesity and decreased risk of Aβ positivity only in the MH group (CU: OR = 0.38, 95% CI 0.16–0.90; MCI: OR = 0.38, 95% CI 0.23–0.63) (Supplementary Fig. 3). The interaction between obesity and metabolic health for Aβ positivity showed statistical significance in the MCI group (*p* for obesity × metabolic health status = 0.002) and borderline significance in the CU group (*p* for obesity × metabolic health status = 0.052). In the mediation analyses, in MCI group, the association between obesity and higher HV as well as higher MMSE scores were fully mediated by the presence of Aβ positivity (Supplementary Fig. 4). However, since the distribution of hippocampal volumes and MMSE scores in the CU group was skewed, it might be inappropriate to proceed mediation analyses in the CU group. This might be related to the lack of variance in the HV or MMSE scores in CU group.

No significant interaction was observed between age, obesity, and metabolic health in patients with Aβ positivity (*p* for age × obesity × metabolic health status = 0.941).

## Discussion

In this study, we systematically investigated the effects of BMI on AD and CSVD markers, in relation to metabolic health, within a large Asian cohort without dementia. Our major findings are as follows: Firstly, obesity was associated with a reduced risk of Aβ positivity. Notably, the protective effects of obesity on Aβ positivity were particularly pronounced in the MH group compared to the MU group. Secondly, being underweight was associated with a higher risk of Aβ positivity. However, contrary to our expectations, no differences were found in the effects of being underweight on AD markers according to metabolic health status. Finally, obesity, but not being underweight, was predictive of severe WMH. Metabolic health did not affect the relationship between obesity and severe WMH. Taken together, our findings have uncovered novel associations between BMI and AD as well as CSVD markers, taking into account metabolic health. These results underscore the importance of adopting robust strategies to maintain an appropriate weight and metabolic health to mitigate the risk of AD pathology and related cognitive decline.

Our conclusion which highlights the favorable effects of obesity in the context of a MH status on clinical outcomes through AD-related pathways (but not CSVD), finds support in the following observations: (1) obesity in MH condition is associated with decreased Aβ positivity, but not with having WMH; (2) decreased Aβ positivity mediate the relationship between obesity in MH condition and higher HV and MMSE scores; (3) obesity in a MH condition is predictive of slower cognitive decline. Most previous studies did not consider the effects of metabolic health on the relationships between BMI status and cognitive impairments. However, our conclusion is supported by a previous study conducted participants from the Alzheimer’s Disease Neuroimaging Initiative mainly including non-Hispanic whites (NHWs), which demonstrated that metabolically healthy obesity (MHO) was associated with decreased Aβ burdens and a reduced ratio of conversion to dementia [21].

Furthermore, emphasizing the significance of our conclusion is crucial, particularly in light of the observed ethnic variations (NHWs and Asians) in the effects of BMI on the MU condition. Specifically, Asians tend to have higher visceral fat and lower subcutaneous fat compared to NHWs [38], which might be associated with a higher prevalence of cardiometabolic syndrome and its complications in Asians compared to NHWs [15]. Thus, our findings could help reduce knowledge gaps in our current understanding of the association between obesity in the context of a MH condition and clinical outcomes, in the context of Aβ positivity, across different racial/ethnic populations.

The mechanisms underlying the beneficial effects of obesity in the context of a MH status on AD markers remain to be elucidated. However, these mechanisms may be attributed to differences in body composition and fat distribution between the MH and MU groups. That is, obese individuals in the MH group may have a more favorable body composition, including higher muscle mass, higher subcutaneous fat, and lower visceral fat compared to those in the MU group. More specifically, a more favorable body composition is closely related to a higher level of adiponectin, which is associated with decreased Aβ burden and neuroinflammation [39]. In fact, previous studies from our group suggested that higher muscle mass and subcutaneous fat mass were negatively correlated with Aβ uptakes [40]. In addition, an increased waist-to-hip ratio was predictive of decreased cortical thickness [41].

We have also found new relationships between being underweight, AD markers and clinical outcomes; being underweight is associated with higher Aβ positivity which in turn leads to lower HV and MMSE. Our findings regarding the association between being underweight and higher Aβ positivity are consistent with those of previous studies [1–3]. Several mechanisms could explain this association. First, the underweight population has decreased levels of insulin-like growth factor 1(IGF-1), which plays a key role in anti-inflammatory responses [42, 43]. Alternatively, given that being underweight is related to sarcopenic status [44], and sarcopenia leads to an increased systemic inflammatory reaction, might explain the link to neuroinflammation [45]. However, contrary to our expectations, metabolic health did not appear to affect these relationships. The powerful effects of being underweight on AD markers may override the effects of MU conditions on AD markers. Since the exact mechanism of this finding was not fully understood, further experimental studies on biological substances such as IGF-1 and adipokines could add valuable insights into the complex relationship between BMI status, metabolic health status, and AD.

Unlike AD markers, obesity (but not being underweight) has an adverse effect on severe WMH. Metabolic health did not seem to affect the relationship between obesity and severe WMH. Our findings might be explained by previous studies showing that MHO individuals have a borderline risk of coronary heart disease [46, 47]. Furthermore, previous studies have suggested that MHO individuals have an increased risk of stroke compared to those with normal weight [48–50]. Interestingly, in this study, WMH did not mediate the relationship between obesity and clinical outcomes. Thus, our results suggest that body weight control should be tailored according to metabolic health status. In other words, in a MH condition, obesity has protective effects against Aβ accumulation and therefore does not need to be tightly controlled. However, in the MU condition, obesity may not only contribute to the development of cardiovascular disease but may also increase WMH burdens in the brain, so a tighter control of body weight is needed.

A key strength of this study is that we systematically investigated the relationship between BMI and AD as well as CSVD markers in relation to metabolic health in a large cohort of Asian individuals without dementia. However, our study has several limitations that warrant further discussion. First, it is important to acknowledge that, as a metric, BMI does not provide additional insights into body composition, such as muscle mass and visceral fat distribution. Therefore, future studies should consider the use of metrics that capture information on body composition. Second, both BMI and metabolic health are dynamic conditions that change continuously throughout an individual’s lifetime [51]. Thus, further investigation into longitudinal changes in BMI and metabolic health and their impact on AD is required. Third, the number of individuals in the underweight group was relatively small. Further studies with a larger number of underweight participants are required to investigate the clinical effects of being underweight. Finally, the generalizability of this study to community-based populations is limited due to the cohort being recruited from a memory clinic setting, which tends to attract a more “health-seeking” demographic. Nonetheless, the findings are relevant as they reflect scenarios commonly encountered in clinical practice. Importantly, this study provides a comprehensive analysis of the association between BMI and various markers of AD and CSVD related to metabolic health.

In conclusion, obesity was associated with lower risk of Aβ positivity only in MH group, while being underweight was associated with higher risk of Aβ positivity regardless of metabolic health status. Furthermore, obesity in MH group were predictive of increased hippocampal volume and better cognitive performances through lower risk of Aβ positivity. Finally, obesity in MH group was related to slower longitudinal cognitive decline. Therefore, our findings underscore the importance of implementing robust strategies aimed at maintaining both appropriate weight and metabolic health to mitigate the risk of AD pathology and the associated cognitive decline.

## Electronic supplementary material

Below is the link to the electronic supplementary material.

Supplementary Material 1

Supplementary Material 2

## Data Availability

Anonymized data for our analyses presented in the present report are available upon request from the corresponding authors.

## Abbreviations

Aβ: amyloid beta
AD: Alzheimer’s disease
BMI: Body mass index
CI: Confidence interval
CREDOS: Clinical Research Center for Dementia in South Korea
CSVD: Cerebral small vascular disease
CU: Cognitively unimpaired
FBB: ^18^F-florbetaben
FLAIR: Fluid-attenuated inversion recovery
FMM: ^18^F-flutemetamol
HDL-C: High-Density Lipoprotein Cholesterol
HV: Hippocampal volume
IDF: International Diabetes Foundation
IGF-1: Insulin-like growth factor 1
MCI: Mild cognitive impairment
MH: Metabolically healthy
MHO: Metabolically healthy obesity
MMSE: Mini-Mental State Examination
MRI: Magnetic resonance imaging
MU: Metabolically unhealthy
NHWs: Including non-Hispanic whites
OR: Odds ratio
rdcCL: regional direct comparison Centiloid
SD: Standard deviation
PET: Positron emission computed tomography
WMH: White matter hyperintensities
