# Correction: Different associations between body mass index and Alzheimer’s markers depending on metabolic health

**DOI:** 10.1186/s13195-024-01625-2

**Published:** 2024-12-02

**Authors:** Eun Hye Lee, Heejin Yoo, Young Ju Kim, Bo Kyoung Cheon, Seungho Ryu, Yoosoo Chang, Jihwan Yun, Hyemin Jang, Jun Pyo Kim, Hee Jin Kim, Seong-Beom Koh, Jee Hyang Jeong, Duk L. Na, Sang Won Seo, Sung Hoon Kang

**Affiliations:** 1 Department of Neurology, Samsung Medical Center, Sungkyunkwan University School of Medicine, 81 Irwon-ro, Gangnam-gu, Seoul, 06351 Republic of Korea; 2 Alzheimer’s Disease Convergence Research Center, Samsung Medical Center, Seoul, Republic of Korea; 3 Center for Cohort Studies, Total Healthcare Center, Kangbuk Samsung Hospital, Sungkyunkwan University School of Medicine, Seoul, Republic of Korea; 4 Department of Neurology, Soonchunhyang University Bucheon Hospital, Gyeonggi-do, Republic of Korea; 5 Department of Neurology, Seoul National University Hospital, Seoul National University college of Medicine, Seoul, Republic of Korea; 6 Department of Digital Health, SAIHST, Sungkyunkwan University, Seoul, Republic of Korea; 7 Department of Health Sciences and Technology, SAIHST, Sungkyunkwan University, Seoul, Republic of Korea; 8 Department of Neurology, Korea University Guro Hospital, Korea University College of Medicine, 148 Gurodong-ro, Guro-gu, Seoul, 08308 Republic of Korea; 9 Department of Neurology, Ewha Womans University Seoul Hospital, Ewha Womans University College of Medicine, Seoul, Republic of Korea; 10 Department of Intelligent Precision Healthcare Convergence, Sungkyunkwan University, Suwon, Republic of Korea

**Correction: Alz Res Therapy 16, 194 (2024)**

10.1186/s13195-024-01563-z

Following the publication of the original article [1], the authors corrected an error in one of the funding institutions under Funding section.

The incorrect funding institution/number:

the “National Institute of Health” research project (2021-ER1006-01).

The correct funding institution/number:

the “Korea National Institute of Health” research project (2024-ER1003-00).

The original article [1] has been updated.

## References

[1] Lee EH, Yoo H, Kim YJ, et al. Different associations between body mass index and Alzheimer’s markers depending on metabolic health. Alz Res Therapy. 2024;16:194. 10.1186/s13195-024-01563-z. 10.1186/s13195-024-01563-z PMC11363444 39210402

