# Supplementary material for: Different associations between body mass index and Alzheimer’s markers depending on metabolic health
Source: Alzheimers Res Ther. 2024 Aug 29;16:194. doi: 10.1186/s13195-024-01563-z (PMC11363444; doi:10.1186/s13195-024-01563-z)
Supplement: Supplementary file 1 — Supplementary Material 1 [file 13195_2024_1563_MOESM1_ESM.docx]

**Supplementary Methods**

**Comprehensive neuropsychological test battery**

We utilized the 2nd edition of the Seoul Neuropsychological Screening Battery (SNSB-II)

that is a well validated and the most common comprehensive neuropsychological test battery in Korea used in the clinical practice [1].

The battery is divided into 5 cognitive domains: attention, language and related functions, visuospatial functions, memory, and frontal/executive functions. Subtests for each cognitive domain are as follows: Vigilance Test, Digit Span Test-forward and backward, Letter Cancellation Test for attention domain; Spontaneous Speech, Comprehension, Repetition, Korean-Boston Naming Test, Reading, Writing, finger naming, right-left orientation, body part identification, calculation, buccofacial praxis, ideomotor praxis for language and related functions; Rey Complex Figure Test-copy and Clock Drawing Test for visuospatial functions; Seoul Verbal Learning Test-immediate recall, delayed recall and recognition, Rey Figure Copy Test- immediate recall, delayed recall and recognition for memory domain; motor impersistence, contrast program, go-no-go, fist-edge-palm, alternating hand movement, alternating square & triangle, Luria loop, Controlled Oral Word Association Test-semantic and phonemic, Korean-Color Word Stroop Test-word reading and color reading, Korean Trail Making Test for frontal/executive functions.

The standardization of the SNSB-II was done with 1,067 subjects (600 males, 467 females) recruited from 6 regions across the country [2]. The age range was 45–90 years old and divided into 9 groups at 5-year intervals. The level of education was classified into seven categories, ranging from illiteracy to 17 or more years of formal education. The SNSB-II provided an enhanced set of norms and indicators through a model-based standardization procedure, whereby the mean and standard deviation for each subgroup are adjusted based on age and education level. This guarantees the stability of normative data trends across age and educational levels, thus circumventing the occasional reversals observed in the original SNSB.

**Aβ positron emission tomography acquisition and quantification**

Three-dimensional positron emission tomography (PET) images were reconstructed using an ordered subset expectation-maximization algorithm with a matrix size of 128 × 128 × 48 and a voxel size of 2 mm × 2 mm × 3.27 mm. The reconstruction parameters used for 18F-florbetaben (FBB) were iterations = 4 and subsets = 20, and for 18F-flutemetamol (FMM) iterations = 4 and subsets = 20.

We used the regional direct comparison centiloid (rdcCL) method to conduct the normalized quantitative analysis of PET-measured Aβ [3]. The rdcCL method quantitatively assesses Aβ in six brain regions by enhancing our research team’s previously developed dcCL method, which was used for the direct development of FBB and FBB- Aβ PET images. In this study, only the global rdcCL was analyzed, without region of interest comparisons. Preprocessing for rdcCL analysis adhered to the methodology outlined by Klunk et al. [4]. The MR images were co-registered to the MNI-152 template, and the corresponding PET images were co-registered to the MRI images. The PET and MRI images were spatially normalized using the transformation parameters of the SPM9 unified segmentation method for T1-weighted MRIs. The reference region was defined using a whole cerebellum (WC) mask obtained from the GAAIN website. The common cortical target region was obtained by comparing the scans of 25 Aβ PET-positive ADD patients and 18 Aβ PET-negative controls among patients who underwent both FBB and FMM-PET with ligand-to-head. Individual rdcSUVR values in the FMM-FBB global CTX VOI were calculated for all PET images. Finally, the MRI-based regional VOIs were defined by superimposing the FMM-FBB global CTX VOI rule from the AAL atlas. The rdcSUVR values of the FBB-FMM CTX VOI were converted to rdcCL units using a conversion equation [5]. The method for obtaining the global and rdcCL is specifically described in previous papers [3].

**MRI acquisition and quantification**

We acquired standardized three-dimensional T1 Turbo Field Echo and three-dimensional fluid-attenuated inversion recovery (FLAIR) images using a 3.0 T MRI scanner (Philips 3.0T Achieva; Philips Healthcare, Andover, MA, USA), as previously described [6].

The images were processed using the CIVET anatomical pipeline (version 2.1.0). Native MRIs scans were registered to the MNI-152 template by linear transformation [7] and corrected for intensity non-uniformities using the N3 algorithm [8]. The registered and corrected images were divided into white matter, gray matter, cerebrospinal fluid, and background. In addition, the inner and outer surfaces of the cortex were automatically extracted using the marching-cubes algorithm to obtain cortical thickness [9], which is defined as the Euclidean distance between the linked vertices of the inner and outer surfaces. The detailed method for obtaining cortical thickness has been described in a previous study [10]. As we extracted cortical surface models from the MRI volumes transformed into stereotaxic space, cortical thickness was measured in the native space by applying an inverse transformation matrix to the cortical surface and reconstructing it in the native space. To measure the hippocampal volume (HV), we used an automated hippocampal segmentation method using a graph-cut algorithm combined with atlas-based segmentation and morphological opening, as described in a previous study [11].

WMH severity was defined using the WMH visual rating scale, proposed by the Clinical Research Center for Dementia in South Korea. According to our previous study, the presence of severe WMH indicates the severity of CSVD markers, including WMH volume, number of lacunes, and number of microbleeds [12]. WMH in the deep subcortical and periventricular regions on FLAIR images were evaluated by experienced neurologists as reported in the literature [6]. Severe WMH was defined based on the following criteria: (1) WMH of 10 mm or more in the periventricular white matter (caps or rim) and (2) WMH of 25 mm or more (maximum diameter) in the deep white matter, consistent with an extensive white matter lesion or diffusely confluent lesion.

**References**

1. Yeonwook Kang SJ, Duk L. Na. Seoul Neuropsychological Screening Battery, 2nd Edition (SNSB-II). Seoul: Human Brain Research & Consulting Co.; 2012.

2. Ryu HJ, Yang DW. The Seoul Neuropsychological Screening Battery (SNSB) for Comprehensive Neuropsychological Assessment. Dementia and Neurocognitive Disorders. 2023;22(1).

3. Kim SJ, Ham H, Park YH, Choe YS, Kim YJ, Jang H, et al. Development and clinical validation of CT-based regional modified Centiloid method for amyloid PET. Alzheimers Res Ther. 2022;14(1):157.

4. Klunk WE, Koeppe RA, Price JC, Benzinger TL, Devous MD, Sr., Jagust WJ, et al. The Centiloid Project: standardizing quantitative amyloid plaque estimation by PET. Alzheimers Dement. 2015;11(1):1-15 e1-4.

5. Cho SH, Choe YS, Kim HJ, Jang H, Kim Y, Kim SE, et al. A new Centiloid method for (18)F-florbetaben and (18)F-flutemetamol PET without conversion to PiB. Eur J Nucl Med Mol Imaging. 2020;47(8):1938-48.

6. Kang SH, Kim ME, Jang H, Kwon H, Lee H, Kim HJ, et al. Amyloid Positivity in the Alzheimer/Subcortical-Vascular Spectrum. Neurology. 2021;96(17):e2201-e11.

7. Collins DL, Neelin P, Peters TM, Evans AC. Automatic 3D intersubject registration of MR volumetric data in standardized Talairach space. J Comput Assist Tomogr. 1994;18(2):192-205.

8. Sled JG, Zijdenbos AP, Evans AC. A nonparametric method for automatic correction of intensity nonuniformity in MRI data. IEEE Trans Med Imaging. 1998;17(1):87-97.

9. Kim JS, Singh V, Lee JK, Lerch J, Ad-Dab'bagh Y, MacDonald D, et al. Automated 3-D extraction and evaluation of the inner and outer cortical surfaces using a Laplacian map and partial volume effect classification. Neuroimage. 2005;27(1):210-21.

10. Kang SH, Park YH, Kim JP, Kim JS, Kim CH, Jang H, et al. Cortical neuroanatomical changes related to specific neuropsychological deficits in subcortical vascular cognitive impairment. Neuroimage Clin. 2021;30:102685.

11. Kwak K, Yoon U, Lee DK, Kim GH, Seo SW, Na DL, et al. Fully-automated approach to hippocampus segmentation using a graph-cuts algorithm combined with atlas-based segmentation and morphological opening. Magn Reson Imaging. 2013;31(7):1190-6.

12. Noh Y, Lee Y, Seo SW, Jeong JH, Choi SH, Back JH, et al. A new classification system for ischemia using a combination of deep and periventricular white matter hyperintensities. J Stroke Cerebrovasc Dis. 2014;23(4):636-42.
