# Supplementary material for: Different associations between body mass index and Alzheimer’s markers depending on metabolic health
Source: Alzheimers Res Ther. 2024 Aug 29;16:194. doi: 10.1186/s13195-024-01563-z (PMC11363444; doi:10.1186/s13195-024-01563-z)
Supplement: Supplementary file 2 — Supplementary Material 2 [file 13195_2024_1563_MOESM2_ESM.docx]

**
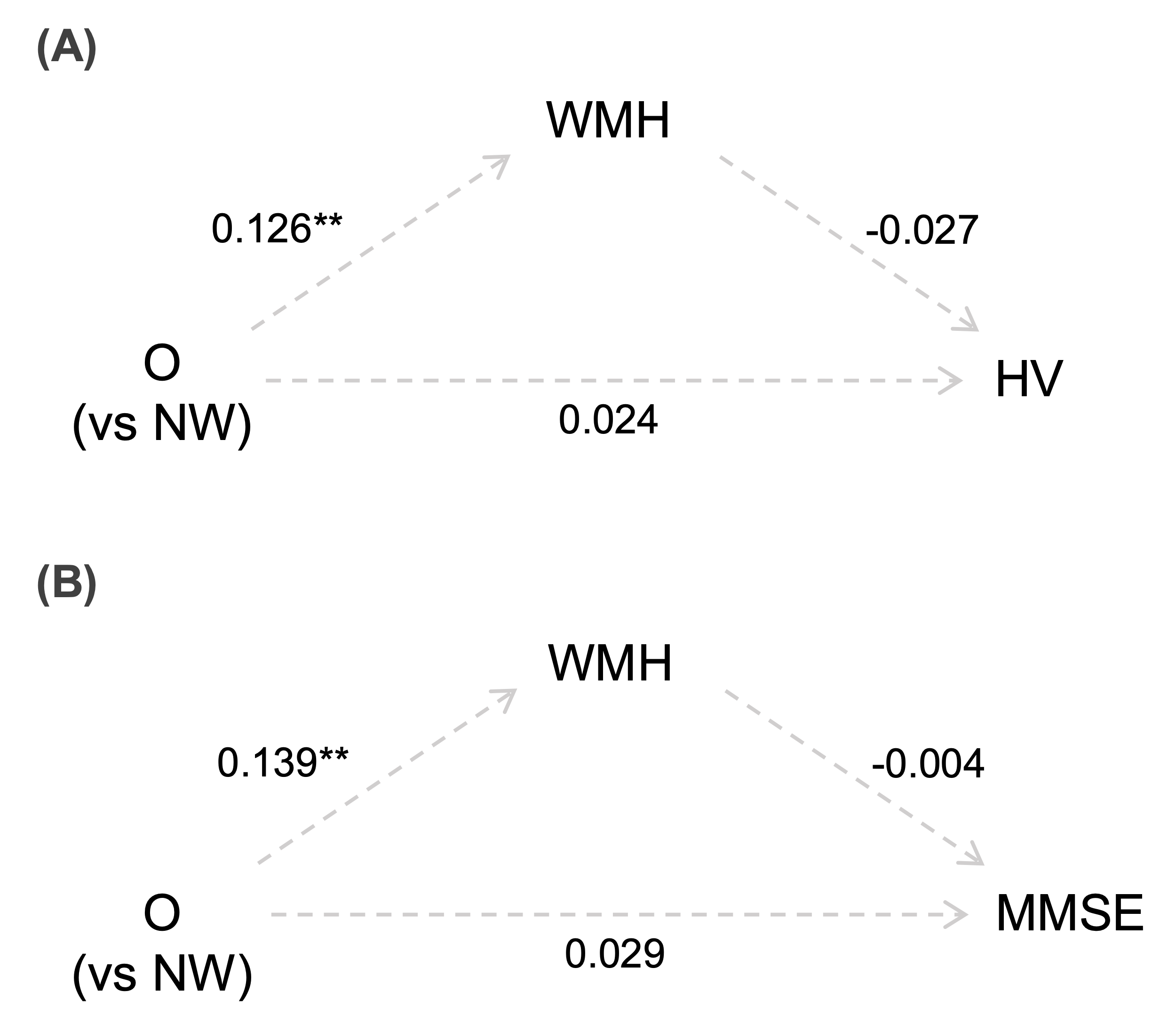
**

**Supplementary Fig. 1** Mediation analyses via severe WMH-mediated pathway. In this study, UW was defined as a BMI < 18.5 kg/m2, NW was defined as a BMI between 18.5 kg/m2 and 24.9 kg/m2, and O was defined as a BMI > 25 kg/m2. Severe WMH did not mediate the relationship between obesity and HV or MMSE scores. Dashed lines indicate associations that were statistically insignificant. β for each association are written on the line.

HV, hippocampal volume; MMSE, Mini-Mental State Examination; NW, normal weight group; O, obese; UW, underweight; WMH, white matter hyperintensity.


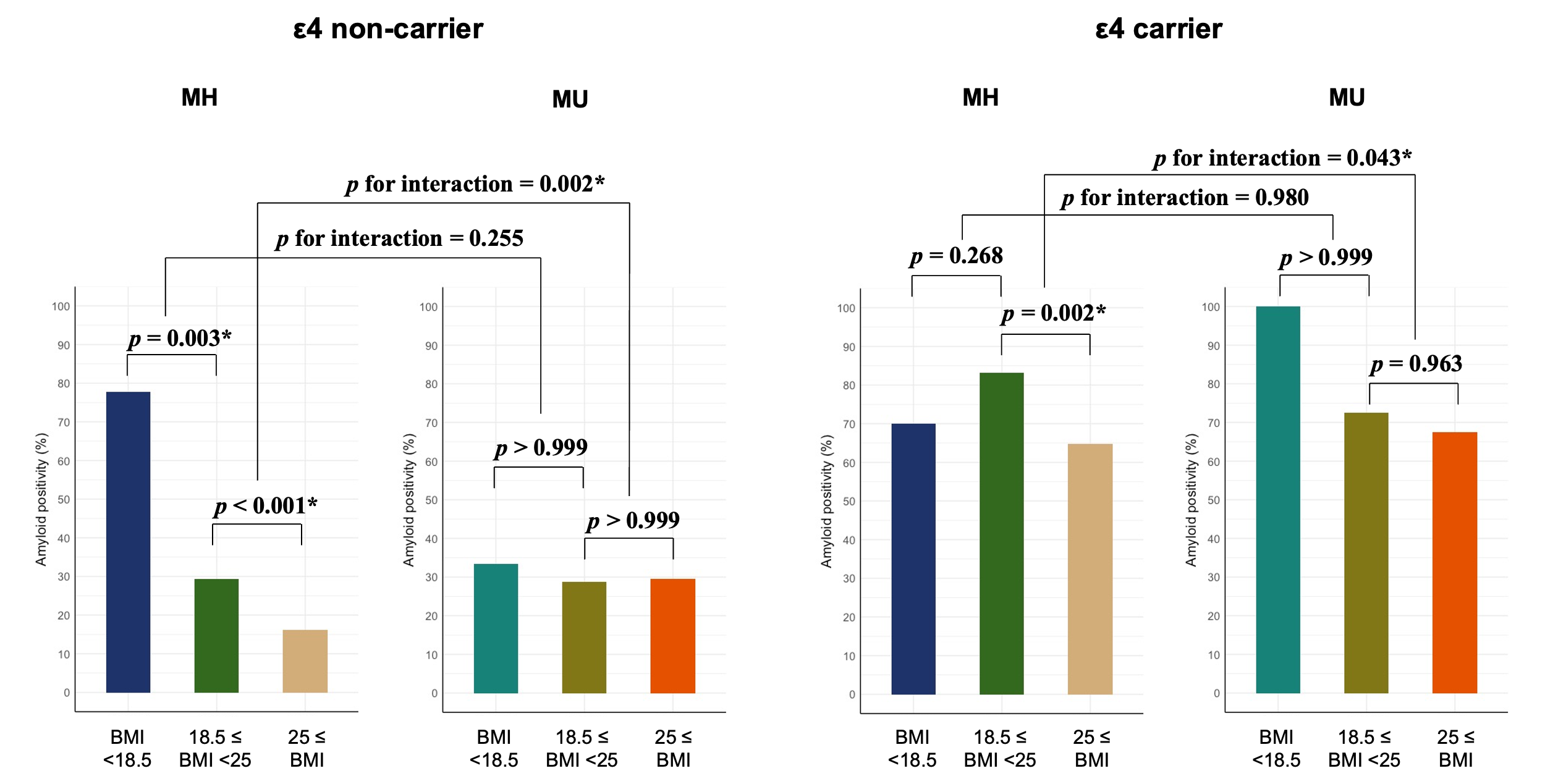


**Supplementary Fig. 2** Effect of metabolic health on the association between BMI status and Aβ positivity or severe WMH in groups stratified by *APOE* genotypes. (A) Obesity was associated with decreased the risk of Aβ positivity only in the MH group regardless of *APOE* genotype groups. There was a significant interaction between obesity and metabolic health on Aβ positivity. (B) No significant interaction was observed between obesity and metabolic health on severe WMH. BMI = body mass index; MH = metabolically healthy; MU = metabolically unhealthy; WMH = white matter hyperintensity.


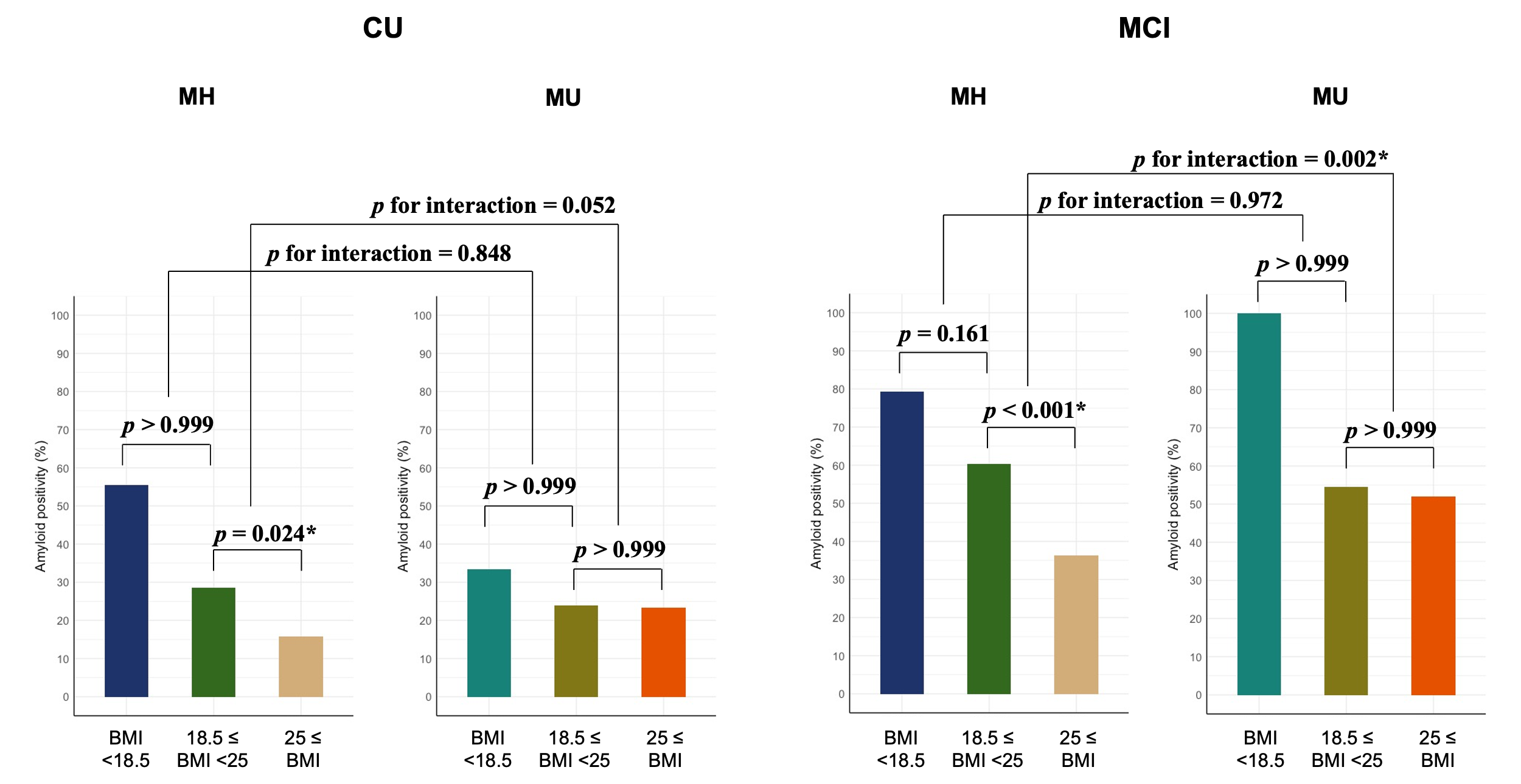


**Supplementary Fig. 3** Effect of metabolic health on the association between BMI status and Aβ positivity or severe WMH in groups stratified by disease stage. (A) Obesity was associated with decreased the risk of Aβ positivity only in the MH group regardless of disease stages. The interaction between obesity and metabolic health on Aβ positivity in the CU group showed borderline significance while there was a significant interaction in the MCI group. (B) No significant interaction was observed between obesity and metabolic health on severe WMH. BMI = body mass index; MH = metabolically healthy; MU = metabolically unhealthy; WMH = white matter hyperintensity.


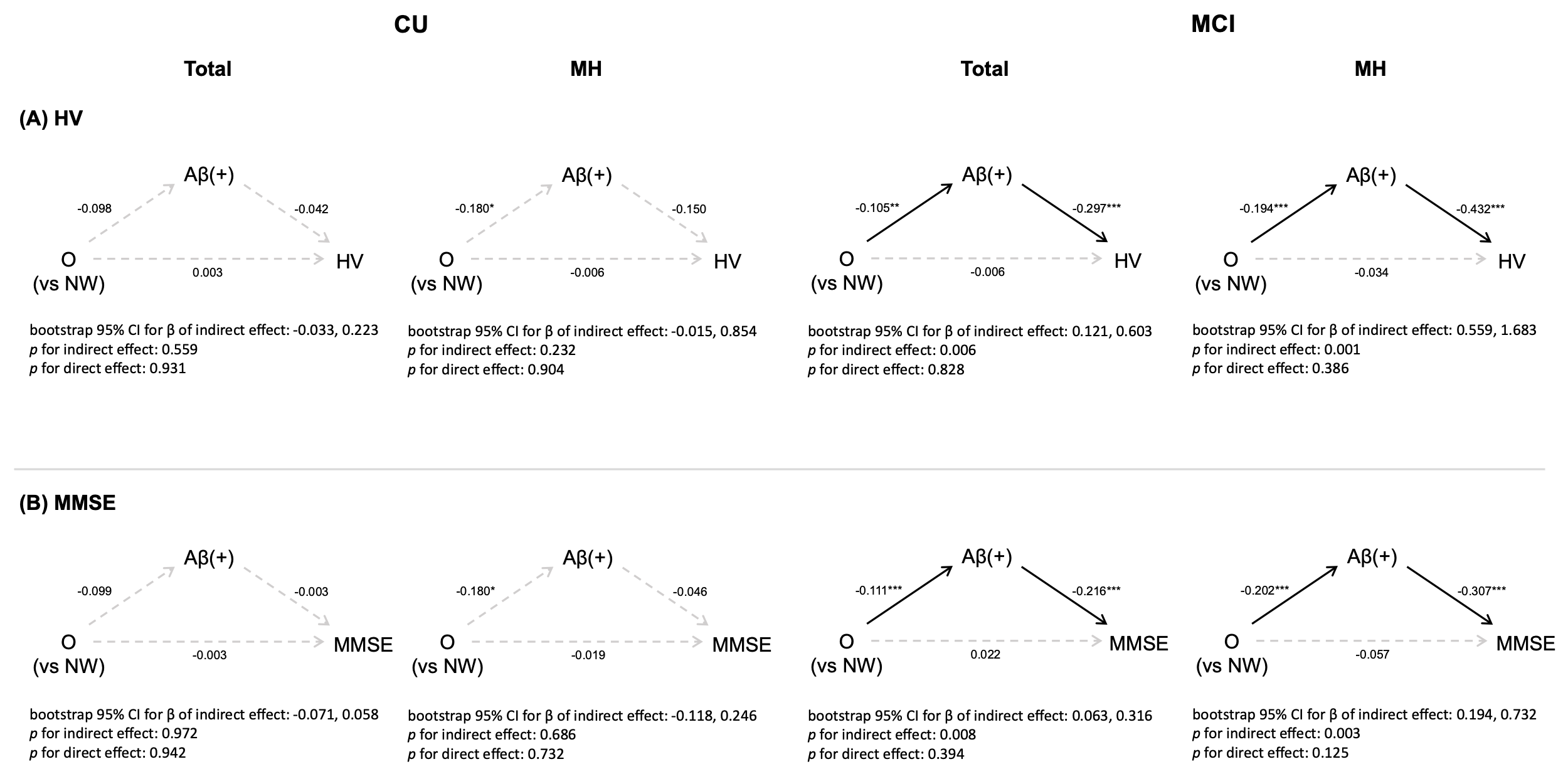


**Supplementary Fig. 4** Mediation analysis via amyloid-mediated pathways in groups stratified by disease stage. (A) There were no significant effects of obesity on HV in the CU group. In the MCI group, Aβ positivity fully mediated the association between obesity and higher HV, both in the total population and in the MH group within the MCI group. (B) There were no significant effects of obesity on MMSE in the CU group. The association of obesity with higher MMSE was fully mediated by Aβ positivity in the total population or in the MH group within the MCI group. In this study, UW was defined as a BMI less than 18.5 kg/m^2^, NW was defined as between 18.5 kg/m^2^ and 24.9 kg/m^2^, and O was defined as a BMI greater than 25 kg/m^2^. Statistically significant associations are expressed as solid lines, whereas non-significant associations are indicated by dashed lines. β value for each association are written on the line. Aβ(+) = amyloid beta positivity; HV = hippocampal volume; MMSE = mini-mental state exam; NW = normal weight group; O = obesity; UW = underweight.
